# Chinese Health Insurance in the Digital Era: Bibliometric Study

**DOI:** 10.2196/52020

**Published:** 2024-07-23

**Authors:** Zhiyuan Hu, Xiaoping Qin, Kaiyan Chen, Yu-Ni Huang, Richard Szewei Wang, Tao-Hsin Tung, Yen-Ching Chuang, Bing-Long Wang

**Affiliations:** 1 School of Health Policy and Management Chinese Academy of Medical Sciences & Peking Union Medical College Beijing China; 2 Department of Education Peking Union Medical College Hospital Chinese Academy of Medical Sciences & Peking Union Medical College Beijing China; 3 College of Medical and Health Science Asia University Taichung Taiwan; 4 Affiliation Program of Data Analytics and Business Computing Stern School of Business New York University New York, NY United States; 5 Evidence-based Medicine Center Taizhou Hospital of Zhejiang Province affiliated to Wenzhou Medical University Taizhou China; 6 Business College Taizhou University Taizhou China

**Keywords:** telemedicine, health insurance, internet plus healthcare, bibliometric, VOSviewer

## Abstract

**Background:**

China has entered the era of digital health care after years of reforms in the health care system. The use of digital technologies in healthcare services is rapidly increasing, indicating the onset of a new period. The reform of health insurance has also entered a new phase.

**Objective:**

This study aims to investigate the evolution of health care insurance within the context of telemedicine and Internet Plus Healthcare (IPHC) during the digital health care era by using scientometric methods to analyze publication patterns, influential keywords, and research hot spots. It seeks to understand how health care insurance has adapted to the growing integration of IPHC and telemedicine in health care services and the implications for policy and practice.

**Methods:**

A total of 411 high-quality studies were curated from the China National Knowledge Infrastructure (CNKI) database in the Chinese language, scientometric analysis was conducted, and VOSviewer software was used to conduct a visualized analysis of keywords and hot spots in the literature.

**Results:**

The number of articles in this field has increased notably from 2000 to 2022 and has increased annually based on a curve of y=0.332exp (0.4002x) with *R*^2^=0.6788. In total, 62 institutions and 811 authors have published research articles in the Chinese language in this field. This study included 290 keywords and formulated a total of 5 hot-topic clusters of “telemedicine,” “IPHC,” “internet hospital,” “health insurance payments,” and “health insurance system.”

**Conclusions:**

Studies on the application of digital technologies in health care insurance has evolved from foundational studies to a broader scope. The emergence of internet hospitals has showcased the potential for integrating IPHC services into insurance payment systems. However, this development also highlights the necessity for enhanced interregional coordination mechanisms. The reform of health insurance payment is contingent upon ongoing advancements in digital technology and increased investment in electronic medical records and primary health care services. Future efforts should focus on integrating technology with administrative systems, advancing mobile health care solutions, and ensuring interoperability among various payment systems to improve efficiency and standardize health care services.

## Introduction

The Chinese government promulgated a policy document regarding the health insurance system in the last few days of the year in 1998 [1,2] and is currently working on establishing the urban employee’s basic health insurance system, which was initiated in 1999 and completed by the end of 1999, and amendment work has continued for several years thereafter [3]. Since then, China’s current concept of a health insurance system has been established.

During the COVID-19 pandemic, the Chinese government has published numerous policies regarding Internet Plus Healthcare (IPHC)–related health insurance services. The policies are mostly guidance documents related to health insurance payment for IPHC services, including web-based health services and telemedicine health services.

A document aiming at promoting the payment of IPHC in health insurance [4] released by China National Healthcare Security Administration in October 2020 indicated that local governments should design and manage the signing of health insurance agreements for IPHC services. Other official arrangements include improving health insurance payment policies, expanding pilot projects, handling health insurance management, and strengthening supervision measures for newly included health insurance health care services among other measures.

As mentioned in a long-term planning official document published by the government of China in December 2022, the importance of IPHC pricing and implementing appropriate services into the health insurance payment list were addressed [5].

IPHC is a novel application of the internet in the health care industry, which includes health education, medical information queries, web-based disease consultations, electronic prescriptions, remote consultations, and various remote forms of health care services such as treatment and rehabilitation [6]. In China, IPHC is an emerging health service model with a cross-industry integration and application of ITs, such as mobile internet, cloud computing, big data, and artificial intelligence [7].

Hence, this field of study is advanced and has real-world implications. The number of studies related to IPHC and telemedicine in 2020, especially in China, has considerably increased [8]. For example, according to reports in early 2020, the number of registrations and IPHC and telemedicine users exponentially increased early during the COVID-19 pandemic; in particular, an internet-based health care platform named “WeDoctor” recorded nearly 80 million visits in early February 2020 and offered services nearly 1 million times. Furthermore, “Ping An Good Doctor,” another major internet-based health care platform claimed to have received over 1 billion visits, and the number of new users has been increasing by several folds [9].

From a historical perspective, telemedicine is an early prototype of China’s IPHC services, which was initiated in the 1990s when doctors in China began communicating with medical experts in other countries through emails about complex and difficult clinical cases. After that, with the increasing use of computers and telecommunications for remote medical consultations in various places, China’s National Healthcare Commission issued regulations to specify the order and behavior of medical care in 1999 to regulate medical order and behavior and enable the development of health care and orderly telemedicine consultation work [6].

In addition, the Chinese government has issued the “Healthy China 2030” project in 2016 [10], which first clearly stated its attitude regarding IPHC, proposing to standardize and promote telemedicine networks and IPHC services and to innovate the IPHC services model.

However, there is no literature using bibliometrics methods, which encompasses this field of the use of health care insurance in IPHC services and telemedicine, and the descriptive study and analysis described here would potentially provide an overview of this area.

This study has 3 objectives: to observe the development of health insurance in telemedicine and IPHC and its related fields in the digital era by examining the publication patterns and key clusters of influential keywords in Chinese. We analyzed the hot spots extracted from high-quality publications and articles based on a bibliometric methodology. We also linked them with future comprehensive studies to illustrate the research frontiers and future roadmap of Chinese health insurance in telemedicine and IPHC service enhancement in the digital era.

## Methods

### Overview

The bibliometric methodology used in this study describes the landscape and core topics of research in the field from a perspective of health insurance in the advancement in digital health care in China from 2000 to 2022.

Bibliometrics is a method of information analysis, which measures research trends and knowledge structures in a field of research to obtain quantifiable, objective data [11]. The method has been extensively used to quantitatively analyze academic literature to describe trending topics and contributions of scholars, journals, and countries and help researchers understand the current research trends, distribution, and core topics in a given field [12,13]. VOSviewer has better visualization in network and cluster analysis than other software, and the scientometric graphs conform better to current academic research styles. VOSviewer was developed by Nees Jan van Eck and Ludo Waltman and features a powerful bibliometric maps function that can clearly visualize the network of literature, keywords, authors, etc [14]. Using VOSviewer, we generated diagrams for institutional cooperation, keyword co-occurrence, author cooperation, and author cocitation, and the Chinese-language data are all retrieved from the China National Knowledge Infrastructure (CNKI) database [15].

### Sampling

All articles related to the fields of health insurance in telemedicine and IPHC published from 2000 to 2022 and written in Chinese are included (Multimedia Appendix 1). The reason why the beginning year was set as 2000 is that China did not formally establish its current concept of a health insurance system until 1999 [1,2].

We set the CNKI as the target database and retrieved data from “Chinese Journal Full-text Database” and “Academic Journals” (excluding dissertations, conference proceedings, and newspapers). On April 5, 2023, we selected the “Advanced Search” feature and set the search strategy as follows: “(Topic: telemedicine (exact) OR Topic: internet plus healthcare (exact)) AND (Topic: health insurance (exact) OR Topic: medical security (exact)) AND (year range 2000-2022)” to select studies from 2000 to 2022; this yielded 659 articles.

**Figure 1:**
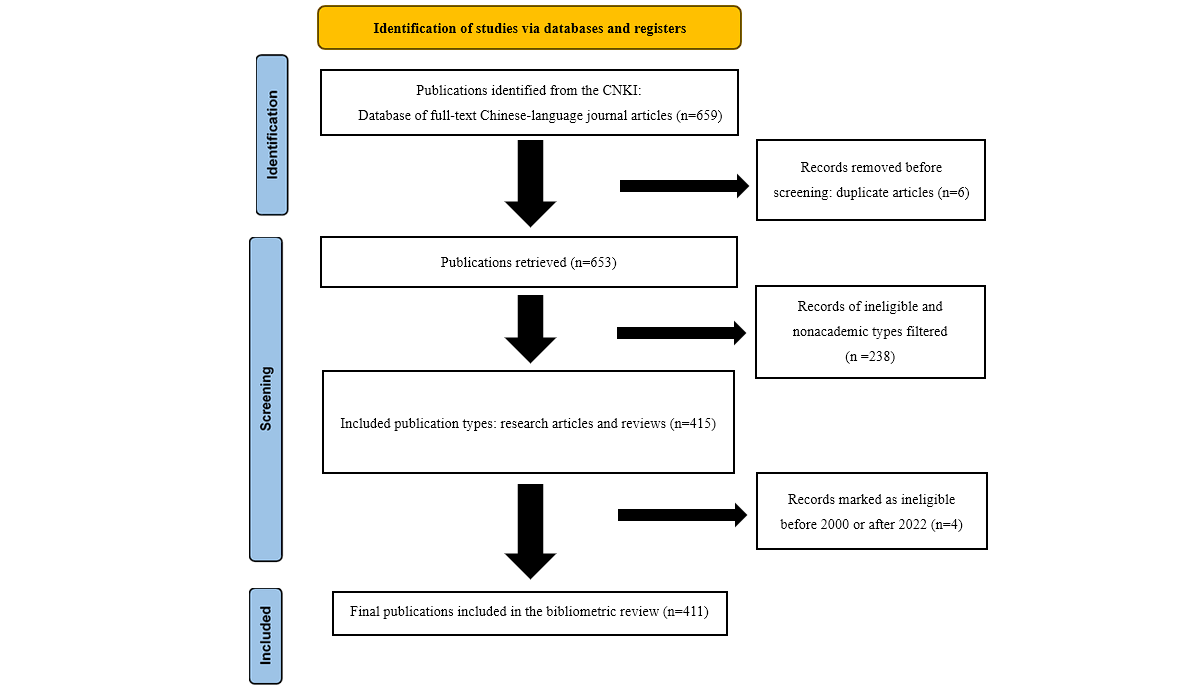
The flowchart for data collection. CNKI: China National Knowledge Infrastructure.

A total of 659 articles retrieved from the search were imported into Excel (Microsoft Corp) for manual checking, and we excluded 6 duplicates papers and then 238 publications belonging to other categories (such as press releases, editorial comments and short reports, nonacademic articles) and 4 publications outside of the time range of 2000-2022 to obtain a final selection of 411 articles; the refinement process is shown in Figure 1. These 411 entries were manually checked to ensure correspondence between the authors and their affiliations, especially when multiple authors are affiliated with the same institution—this step is crucial as it helps avoid potential errors.

We then exported the data and imported them into VOSviewer software (version 1.6.19) for cluster analysis. Based on cluster results, we analyzed and summarized the articles.

## Results

### Publication Trends

Based on the publication year in the research literature, it can be observed that between 2000 and 2015, the number of articles published in the field of IPHC, remote medical services, and health insurance services was <10 each year, and the accelerated growth started in 2015. Since the number of publications approached 14 in 2015, from 2017 to 2019, the number of articles published in this field plateaued around 30 to 40. In 2020, the number of publications in this research field increased rapidly to nearly 100 papers (around 90 a year) and remain high at about 80-90 papers in 2020 and 2021. Index regression predicts that there will still be a number of Chinese studies published in this field in the near future, while the *R*^2^ value of the regression model is 0.6788, indicating that the curve explains the variables relatively well.

### Analysis of the Journals

Table 1 shows that journals whose scope includes health insurance focus more on this research field; *China Health Insurance Journal* accounts for 6.8% of the proportion of studies in this field. Second, journals that explore digital medicine take the lead. *China Digital Medicine* accounts for 4.8% of studies in this regard, followed by other general medical and health policy research journals. Regarding the distribution, core journals included in Chinese core journals indexed by Peking University or the Chinese Social Sciences Citation Index, both of which are top core collections of Chinese-language journals, enjoy widespread prevalence. The top 5 journals are core Chinese-language journals.

**Table 1:** The number of articles in the top 10 journals and their proportions in a total of 411 publications.

Rank	Journal	Articles, n (%)
1	*China Health Insurance*	28 (6.8)
2	*China Digital Medicine*	20 (4.8)
3	*Health Economics Research*	17 (4.1)
4	*Chinese Hospitals*	15 (3.6)
5	*China Social Security*	14 (3.4)
6	*Journal of Medical Informatics*	11 (2.6)
7	*Chinese Journal of Health Informatics and Management*	11 (2.6)
8	*China Health*	9 (2.1)
9	*Modern Hospital*	8 (1.9)
10	*Chinese Health Service Management*	8 (1.9)

### Analysis of the Number of Citations of Articles

This field is in its infancy; hence, the top cited article in this field is a study analyzing the basis of this field. The article containing definitions came first, occupying the forefront, received 201 citations (Table 2). Then, latter research focused on currently established modules, problems faced, and future development trends. They all attempted to establish theories and models needed in this field systematically.

**Table 2:** The top 10 cited Chinese-language articles according to the China National Knowledge Infrastructure.

Rank	Title	Journal	Authors	Citations, n
1	Internet + Medical Mode： Contents and System Architecture	*Chinese Hospital Management*	Zhu Jinsong	201
2	Research on Development Policy of Integration of Medical Care and Pension and Institutional Pension Service for the Elderly	*Medicine and Society*	Ma Lili, Chen Na, and Tang Shaoliang	194
3	The Status Quo of Internet Medical-Based on The Analysis And Investigation on Three Hospitals	*Chinese Journal of Health Policy*	Wang Anqi and Zheng Xueqian	138
4	Analysis on the Development Model of Internet Hospitals in China	*Health Economics Research*	Zhang Mengqian, Wang Yancui, Qian Zhenguang, and Wang Dandan	59
5	Practice and Exploration of Medical Association in Remote Areas	*Modern Hospital Management*	Sun Xizhuo, Gong Fangfang, Gu Xiaodong, Su Qian, and Cai Yutong	55
6	Problems and Countermeasures for “Internet + Healthcare” in China	*Administration Reform*	Luan Yunbo and Tian Zhendu	52
7	Problems and Countermeasures for Medical Service Supply in Elderly Care Institutions from the Perspective of Medical-Old-Age Combination	*Chinese Journal of Gerontology*	Fan Qingmei, Chen Le, Wu Meng, Wu Jiankang, and Li Jiamin	45
8	Analysis on Problems and Countermeasures of Mobile Health Service in China	*Medicine and Philosophy*	Yang Xiaoli and Feng Xinwei	45
9	Study on Regulation System and Related Mechanism of Internet Based Medicine	*Chinese Journal of Health Informatics and Management*	Meng Qun, Yin Xin, and Dong Kenan	44
10	Analysis on Service Mode and Application Status for Network Medical Treatment in China	*Chinese Journal of Health Informatics and Management*	Liu Ning and Chen Min	42

### Analysis of Authors

**Figure 2:**
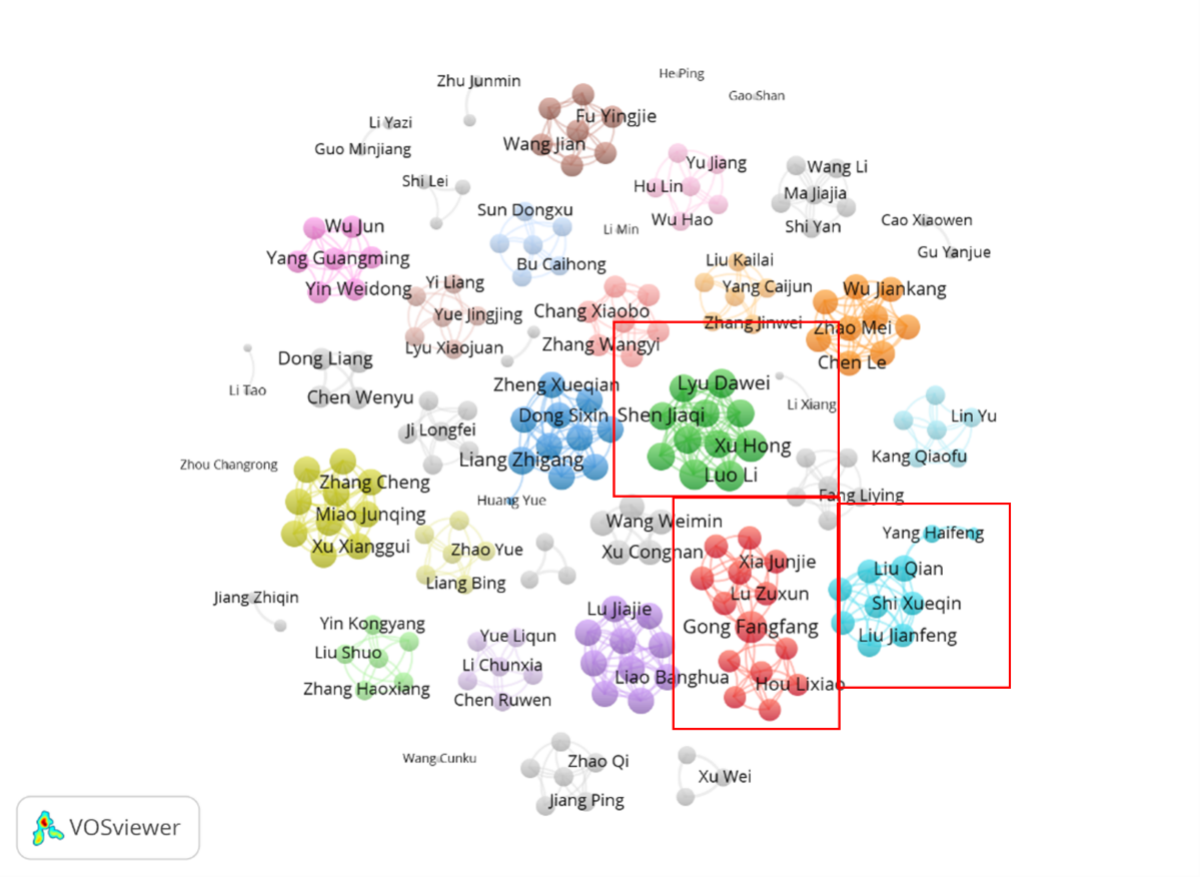
Coauthorship analysis of authors.

Analysis of the author cooperation network revealed that 811 authors had explored health insurance in IPHC and telemedicine, of whom 9 have acquired more than 12 total link strengths (Figure 2), namely total link strength (TLS), reflecting the strength of cooperation in bibliographical analysis (Table 3). Xu Hong and Lyu Dawei, who discussed the prospect of IPHC in cooperative development of the Changjiang river delta, are the most active authors in this field. The latter active authors also gained over 10 TLSs.

**Table 3:** Top Chinese authors ranked according to total link strength.

Rank	Author	Total link strength
1	Xu Hong	16
2	Lyu Dawei	16
3	Zheng Xueqian	14
4	Luo Li	14
5	Wang Weijun	14
6	Liu Qian	14
7	Gong Fangfang	13
8	Sun Xizhuo	13
9	Liang Zhigang	12

### Analysis of Institutions

**Figure 3:**
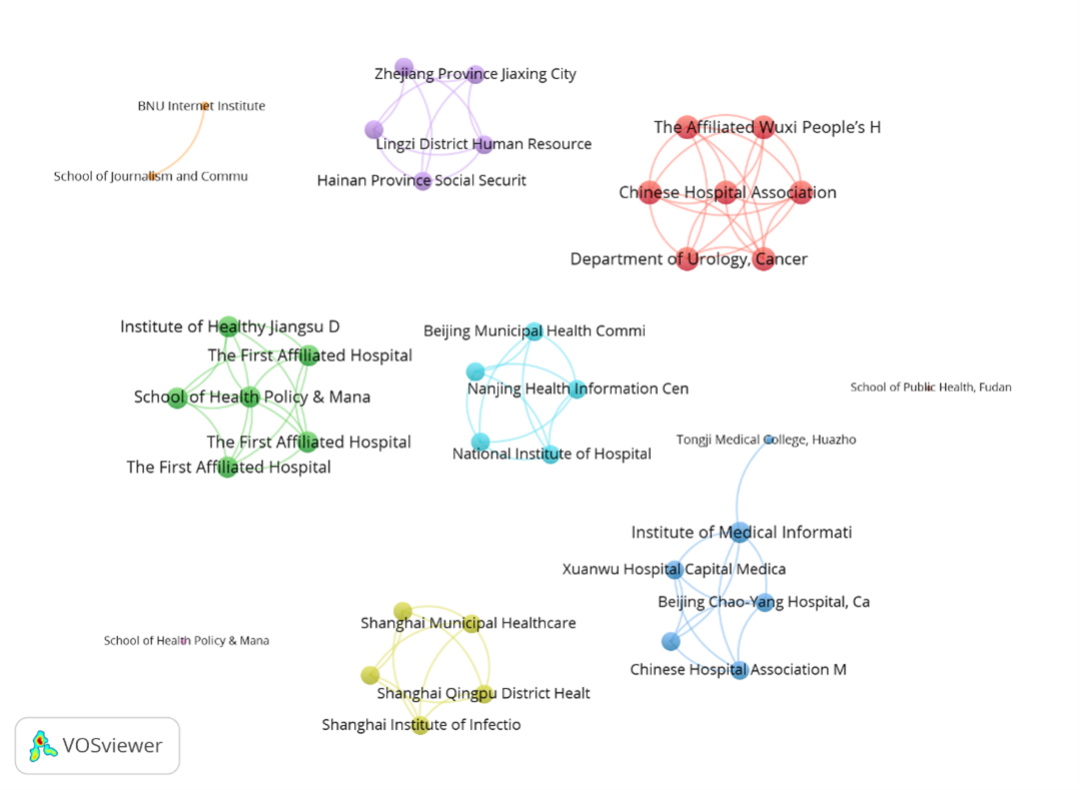
Institution co-occurrence of publications.

A total of 62 institutions were finally included, with a minimum limitation of more than 3 publications, whose publications were analyzed using VOSviewer (Figure 3). Moreover, the School of Health Policy & Management, Nanjing Medical University (TLS=6 times) and other 10 institutions were the top institutions with highest TLS in VOSviewer counting (Table 4). Total link strength and institution co-occurrence of publications.

**Table 4:** Total link strength and institution co-occurrence of publications.

Rank	Institution	Total link strength
1	School of Health Policy & Management, Nanjing Medical University	6
2	Chinese Hospital Association	6
3	Peking University Health Science Center	6
4	Department of Urology, Cancer Hospital Chinese Academy of Medical Sciences	6
5	Beijing Municipal Health Commission	6
6	Nantong University Medical School	6
7	The Second Hospital of Dalian Medical University, Department of Neurosurgery	6
8	Institute of Healthy Jiangsu Development, Nanjing Medical University	6
9	Shanghai Municipal Healthcare Security Bureau	6
10	Shanghai Institute of Infectious Disease and Biosecurity, School of Public Health of Fudan University	6
11	Chinese Hospital Association Medical Legality Specialized Committee	6

### Analysis of Co-Occurrence of Keywords in Chinese

**Figure 4:**
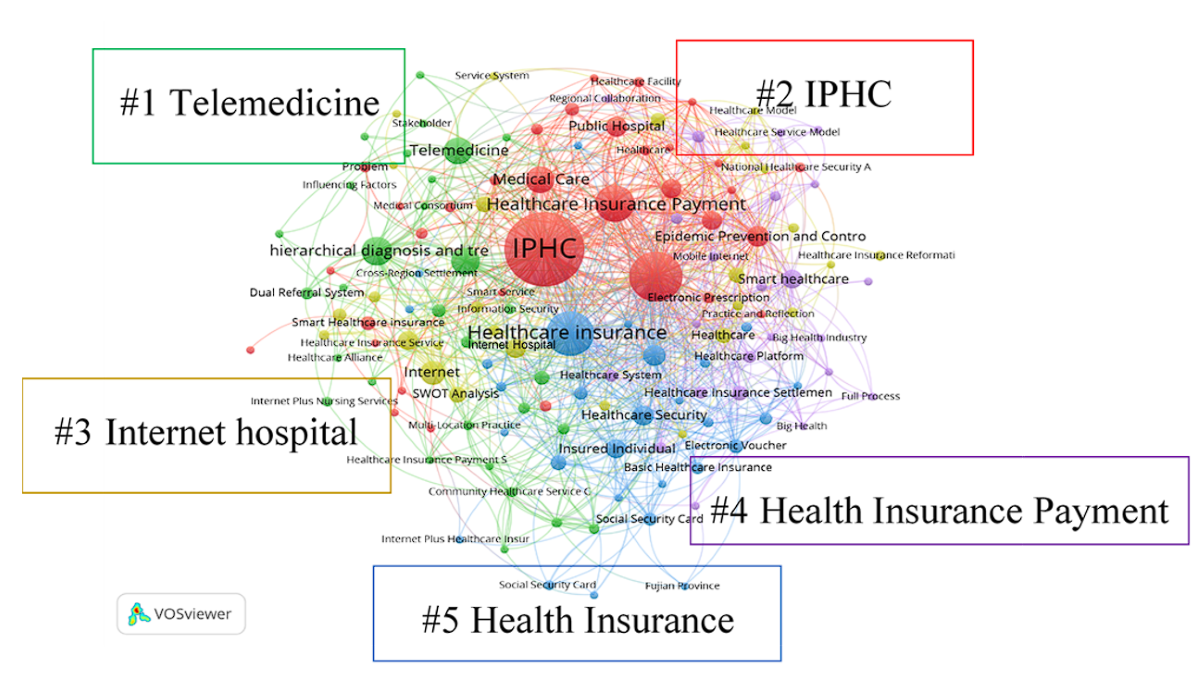
Visualization of keyword co-occurrence analysis. IPHC: Internet Plus Healthcare.

Identifying trending research fields and directions through keyword co-occurrence analysis is an important indicator for monitoring the development of a discipline. Mapping of keywords is shown in Figure 4, where the size of the node represents the frequency of keyword occurrence, and the lines between the nodes reflect the co-occurrence relationships among multiple keywords. According to the mapping image generated by VOSviewer, current hot topics in Chinese literature in this field can be visually described.

This study analyzed 290 Chinese-language keywords that appeared at least 10 times across included publications using VOSviewer. The results were grouped into 5 clusters: “Telemedicine,” “Internet hospital,” “Internet Plus Healthcare (IPHC),” “Health Insurance Payment,” and “Health Insurance system.” These clusters provide insight into the most prominent topics related to the use of health insurance in IPHC and telemedicine (Table 5).

**Table 5:** Main keywords translated to English and their total link strength (TLS).

Keywords		TLS
**Cluster 1: telemedicine**		
	hierarchical diagnosis and treatment	109
	telemedicine	102
	healthcare alliance	81
**Cluster 2: IPHC^a^**		
	internet healthcare	129
	healthcare service	68
	healthcare insurance fund	79
**Cluster 3: internet hospital**		
	internet hospital	179
	smart healthcare insurance	29
	big data	56
**Cluster 4: insurance payment**		
	online payment	72
	healthcare	31
	smart healthcare	29
**Cluster 5: health insurance**		
	national health security administration	41
	insured individual	53
	social security card	41

^a^IPHC: Internet Plus Healthcare.

### Primary Findings From the Co-Occurrence of Keywords

Based on the 5 keyword clusters, the hot spots in the field are described below.

#### Cluster 1

The primary keyword is “telemedicine” and also includes “remote consultation,” “hierarchical diagnosis and treatment,” “healthcare alliance,” etc, focusing on doctor-to-doctor telemedicine services. Also, it includes topics such as the interactions among different health care service providers under remote conditions, remote pathological analysis, remote consultation, and other telemedicine services based on new-style intelligent communication technology [16]. There are more stakeholders in this type of health care service, demanding that health insurance policies be more detailed to take into account real-world situations.

#### Cluster 2 

The primary keyword is “Internet plus healthcare” (IPHC). Also, it includes keywords such as “Internet healthcare,” “Healthcare service,” “health management,” “health insurance reimbursement,” and “health insurance fund.” In 2019, the National Healthcare Security Administration officially launched the construction of a national unified health care security digitalization platform [17]. In 2021, the system was gradually implemented, and the number of designated medical institutions covered by the basic medical cross-provincial and interregional settlement insurance platform is increasing [17]. The construction of digitalized hospitals has better promoted the development of internet health insurance operations.

#### Cluster 3

The primary keyword is “internet hospital.” Also, it includes keywords such as “healthcare service,” “online healthcare,” “smart healthcare insurance,” “electronic prescriptions,” and “smart healthcare.” It mainly focuses on providing health management services for patients with chronic disease on the web, especially under lockdown policies for epidemic prevention and control [18]. Furthermore, as an impact of the COVID-19 epidemic, health care service units in various regions have embraced the internet and provided web-based consultation and diagnostic services. Some researchers also involved health insurance payment [17]. In the future, similar methods can be used to provide older adult–focused health care services for the aging population [19].

#### Cluster 4

The primary keyword is “health insurance payments,” and it also includes “online payments,” “health insurance payment reform,” “insured population,” and “health insurance files.” The research and analyses are mainly policy-oriented, focusing on reducing the burden of health insurance funds and developing reasonable health care service prices and the comparison of different actual implementations of web-based insurance payments in different provinces and municipalities, as well as the future development of smart insurance [20].

#### Cluster 5

The primary keyword is “health insurance system” and involves keywords such as “medical service prices,” “health insurance funds,” and keywords such as “National Healthcare Security Administration,” and “designated hospitals for health insurance.” The studies and analyses mainly focus on the systematic constructions for health insurance services, the modernization and innovation of the health insurance system from a macro view in the new digital era [21], and the necessary adjustments and changes required for the system to adapt to the new pattern of health care services landscape through the digital era.

## Discussion

### Principal Findings

Our study shows that in the digital era, China’s health care service system is facing the need for payment mechanisms and policy adjustments to support and optimize the hierarchical health care system. The practice of internet hospitals, as a part of IPHC, has demonstrated the potential for insurance payment in web-based health care services, but it has also revealed challenges in regional integration and interregional coordination. Insurance payment reform, as a key lever for driving systemic change, relies on the advancement of digitalization and informatization, as well as continuous investment in electronic medical records, IT, and primary health care services. Future research and policy making must focus on addressing the integration of technologies with administrative systems, promoting the development of mobile health care, and exploring the interoperability between health care insurance payment systems to achieve efficiency and standardization in health care services.

According to our results, there has been significant development in areas of health insurance in IPHC and telemedicine research over the past 2 decades. The number of relevant publications has steadily increased year by year, and more than 60 researching institutions and over 800 authors in China have published academic research papers in this field. Since 2017, the number of publications in this field has risen sharply from around 30 papers each year to around 90 papers each year, which means that in the near future, an increasing number of studies will focus on the use of health insurance in IPHC and telemedicine, especially in improving health insurance implementational methods and measures, and future patching policies regarding digital health care services.

In the founding stage of the research field, the most highly cited paper was an analysis of its theoretical basis, with 201 citations, occupying the top spot, having focused on established modules, current challenges, and future development trends, striving to systematically develop theories and models required for the field.

Among 62 institutions, over 10 of them had a TLS of 6 and rank at the top.

There were 62 institutions with a minimum of >3 publications; among them, 11 institutions with 6 TLSs were the top academic institutions. These research institutions include universities, hospitals, and associations in the health care industry, implying that this research field has received widespread attention in Chinese academic fields and reflects the importance of research in this field in China. Different types of institutions engage in academic discussions from their specific perspectives.

Through bibliometric and visualization analysis, we gained a deeper understanding of the overall landscape of this research field, including prominent Chinese authors and publishing institutions, as well as their collaborative relationships and academic influence. This information provides researchers with transparent channels for selectively obtaining advanced and valuable research results. Co-occurrence analysis can also depict research trends and hot spots [22], providing researchers with assistance in proposing research topics to convince funding agencies to develop more effective funding plans.

### Analysis of Research Focus

#### Overview

Keywords are essential in a research article and contain the most important information [23].

Based on the analysis of keyword clusters in the literature [24], this study focuses on different aspects and levels of research directions that health insurance needs to promote in implementing IPHC, as well as the problems and possible solutions reflected in the actual health care service practice [25]. From the integration of health care services and medical treatment to the connection between health insurance systems, based on the interconnection of information platforms [26], the key conflicts encountered in health care service practice are gradually being resolved and improved [27]. There are a total of 5 clusters, and based on the research results, the main research frontiers involved in these 5 clusters will be discussed below.

#### Cluster 1

The primary keyword is “telemedicine.” By constructing a telemedicine service system between institutions in a medical treatment combination, which involves multiparty participation such as bidirectional referral and web-based consultation [28], the mechanism of health insurance payment needs to be strengthened and improved. Furthermore, to improve the hierarchical health care system, a mixed health insurance payment model should be explored [29]. In the context of combining remote health care services with health insurance, the current emerging problems such as the barriers to the generation of cross-provincial systems need to be clearly defined, and policies need to be adjusted and improved accordingly [30].

#### Cluster 2

The primary keyword is “Internet plus healthcare.” The development of IPHC requires continuous advancement in medical digitalization [31], strong modern digital technologies such as artificial intelligence, digital twins, big data management, and remote services to realize many health care services that are currently only in their infancy, such as smart health care, which are the forefronts of current research [32]. The platform construction of electronic medical records and electronic prescriptions has been integrated with intelligent health care security platforms. However, in certain specific implementation, there are still many practical issues such as technology advancing ahead of management models [33]. Advancing creatively through a combination of technology and policies is necessary to solve many potential new problems and conflicts. In the future, IPHC will remain an important theme for China’s medical and health reforms and development [34], and there will inevitably be more academic studies on policies and management for this topic.

#### Cluster 3

The primary keyword is “internet hospital.” Some internet hospitals have connected with local health insurance individual account payment channels to achieve health insurance reimbursement [35]. The policy enables follow-up services for chronic diseases to be included in the scope of health insurance payment, accomplishing a series of closely matched services such as web-based remote consultation [19], web-based prescription, and health insurance payment for purchasing medication [18]. Health management can also be beneficial, such as improving medication compliance and strengthening awareness of chronic disease treatment and community health management [36]. The inclusion of internet follow-up services in health insurance payment emphasizes the homogenization of offline and web-based diagnosis and treatment behaviors [37], providing a basis for health insurance pricing through the use of advanced technological assessments and other means [20], and requires further research, which is a topic of high interest.

#### Cluster 4

The primary keyword is “health insurance payments.” The use of health insurance in internet-based medical payment is increasing, but problems and difficulties have emerged. For example, there are significant problems in the integration of regional health insurance, internet-based medical platforms, and local health insurance systems, as well as communication and coordination issues such as cross-regional medical treatment and settlement [38]. As one of the main directions for future development of internet-based health care services, the use of mobile health care through smartphones is also a current research hot spot [39].

#### Cluster 5

As the most powerful lever to drive reforms in the entire medical system, health care payment reforms are crucially supported by digital and information-based payment systems, so the IPHC with health insurance occupies an important position [40]. “Electronic medical records” for referral purposes and more sophisticated IT can accelerate the implementation of payment reforms [41]. Vigorous development of primary care–based internet health care [42], increasing the proportion of health insurance expenditures in this area, among other measures. Research frontiers include problems with interoperability among internet health care systems, hospital internal management platforms [43], electronic medical record systems, imaging and inspection platforms [44], and other related issues.

### Strength and Limitations

To the best of our knowledge, no study has carried out bibliometric analysis in this field of research on the use of health insurance in IPHC services, along with telemedicine. It is of noticeable significance to discuss these issues and it is now necessary for us to study the research topic and identify hot spots. The method of bibliometrics and visual analyses enable us to sort out research focuses in recent publications as well as their correlation and differentiation.

However, this study inevitably has some limitations. We only retrieved studies and reviews on research topics related to this field from the CNKI. Although the CNKI plays a significant role in academic research and literature analysis in China, there are some obvious limitations to its application in international research fields, and studies need to consider these limitations and comprehensively integrate database resources in accordance with the specific needs of the research, to ensure comprehensive evaluation and analysis. Therefore, we may have missed some publications due to database limitation, and articles related to other languages may not have been included.

Bibliometric methods provide an overall insight into the landscape of a specific research field, but researchers and policy makers should be aware that the feature is not detailed enough for the evaluation or decision-making, and this study provides no enough in-depth insight into the influential articles and authors in this field. Balanced approaches that integrate bibliometrics research with other assessment methods can provide a thorough understanding of research impact and trends.

### Future Directions

Recently, many previously ignored issues have been discussed and are now at the forefront, and numerous real-world problems associated with internet-based health care services, which has been limited by the scale of services, have been pointed out and discussed. Starting from the postpandemic era, the demand for remote or IPHC services will continue to grow. The deepening support of policies for IPHC services can meet the demand for health insurance management, mobile insurance payment, and cost reduction. Future research hot spots are developing, such as those focusing on the web-based application of live broadcasting, new media, artificial intelligence technology, etc, into IPHC services. Other hot spots include chronic disease management and primary health care, as well as the community older adult care in health insurance reforms in the IPHC era. These hot spots are surely important for suggesting more reasonable policy measures, enhancing the accessibility of health care services, reducing costs, and improving the quality of health care, thus better serving the people requiring them.

In summary, this study on the application of health insurance in internet-based health care services is quite forward-looking, and it is an important frontier for future health care service research.

### Significance

This study describes a bibliometric analysis of the current high-quality Chinese literature on the application of telemedicine and IPHC in health insurance in China. This study used the popular software tool VOSviewer (version 1.6.19) to analyze the literature published in the CNKI, which was involved in the development of this field, and provides an overview of all the existing high-quality Chinese research and guides future research developments to improve the application of telemedicine and IPHC in health insurance. In particular, the unique IPHC paradigm from China is of importance to health care professionals worldwide.

### Conclusions

This study used bibliometric analysis to describe the current situation and trends of health insurance in IPHC and telemedicine from the literature in China. This study highlights prominent research institutions, hospital researchers, and researchers at research institutes in universities engaged in this field. More articles on health insurance in IPHC and telemedicine are expected to be published in the next few years. The use of internet hospitals has underscored the potential of health care insurance payment in IPHC services, but it also highlights challenges pertaining to regional integration and interregional coordination. As the key lever for instigating systemic change, health care insurance payment reforms hinge on the progression of digitalization and informatization, along with ongoing investment in electronic medical records, IT, and primary health care services. Future research and policy formulation are expected to focus on tackling the integration of technologies with administrative systems, fostering the advancement of mobile health care, and delving into the interoperability among health care payment systems to attain efficiency and standardization in medical health care. This research provides an invaluable reference, enhancing our grasp of the current landscape and prospective progress in the field of health insurance within the domains of IPHC and telemedicine.

## Appendix

Multimedia Appendix 1 The number of cumulative publications (2000-2022) and model fitting curve.

## Abbreviations

CNKI: China National Knowledge Infrastructure
IPHC: Internet Plus Healthcare
TLS: total link strength

## Data Availability

All data and material generated or analyzed in this study are included in this published paper.
