# Supplementary figures and images for: Chinese Health Insurance in the Digital Era: Bibliometric Study
Source: Interact J Med Res. 2024 Jul 23;13:e52020. doi: 10.2196/52020 (PMC11303892; doi:10.2196/52020)

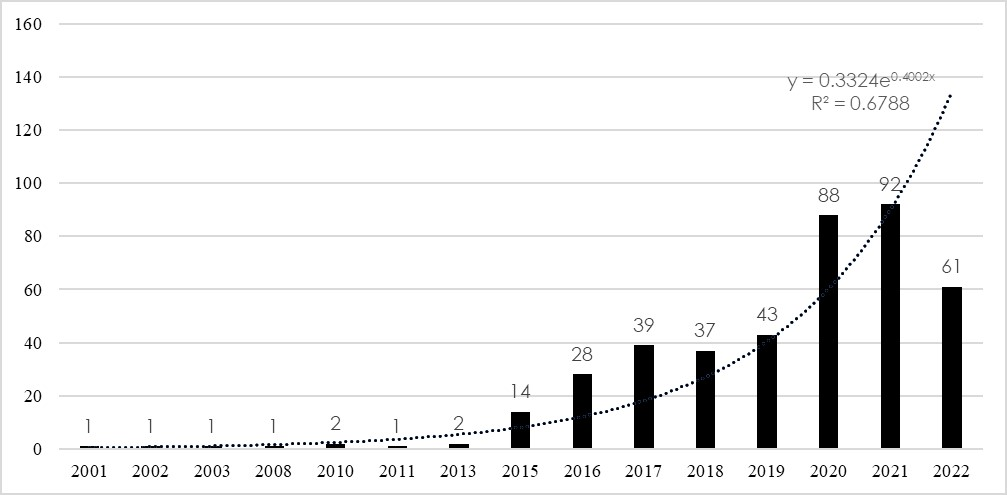

Supplement: Multimedia Appendix 1 [file ijmr_v13i1e52020_app1.png]
